# Shifting management of a community volunteer system for improved child health outcomes: results from an operations research study in Burundi

**DOI:** 10.1186/1472-6963-15-S1-S2

**Published:** 2015-06-08

**Authors:** Jennifer Weiss, Raphael Makonnen, Delphin Sula

**Affiliations:** 1 Concern Worldwide, US, 355 Lexington Ave, 19th Floor New York, NY 10017, USA; 2 Mailman School of Public Health, Columbia University, 722 W. 168th Street, New York, NY 10032, USA; 3 Concern Worldwide, Burundi, INNS Quartier, Muyinga Avenue No. 38, Bujumbura, Burundi

**Keywords:** Child health, behavior change, Community Health Workers, task-shifting, Care Groups, Burundi

## Abstract

**Background:**

Community-based strategies that foster frequent contact between caregivers of children under five and provide credible sources of health information are essential to improve child survival. Care Groups are a community-based implementation strategy for the delivery of social and behavior change interventions. This study assessed if supervision of Care Group activities by Ministry of Health (MOH) personnel could achieve the same child health outcomes as supervision provided by specialized non-governmental organization (NGO) staff.

**Methods:**

The study was a pretest-posttest quasi-experimental design implemented in Burundi. A total of 45 MOH-led Care Groups with 478 Care Group Volunteers (CGVs) were established in the intervention area; and 50 NGO-led Care Groups with 509 CGVs were formed in the comparison area. Data were collected from 593 and 700 mothers of children 0-23 months at baseline and endline, respectively. Pearson’s chi-squared test and difference-in-difference analysis assessed changes in 40 child health and nutrition outcomes. A qualitative process evaluation was also conducted midway through the study.

**Results:**

The MOH-led Care Group model performed at least as well as the NGO-led model in achieving specific child health and nutrition outcomes. Mothers of children 0-23 months in the intervention and comparison sites reported similar levels of knowledge and practices for 38 of 40 dependent variables measured in the study, and these results remained unchanged after accounting for differences in the indicator values at baseline. Process monitoring data confirmed that the MOH-led Care Group model and the NGO-led Care Group model were implemented with similar intervention strength.

**Conclusions:**

The study demonstrated that behavior change interventions traditionally led by NGOs can be implemented through the existing MOH systems and achieve similar results, thereby increasing the potential for sustainable child health outcomes. Future research on the MOH-led Care Group model is required to systematically document all inputs and monetary costs borne by the MOH to implement the model.

## Background

Strengthening community health systems through a well-trained and supported workforce is an important step to reducing child mortality, addressing inequity, and attaining universal health coverage [1,2]. As a critical cadre of the health workforce, Community Health Workers (CHWs) have made significant contributions towards child survival priorities in several low income countries [3]. There is substantial evidence documenting CHWs’ effectiveness in generating demand for health services and expanding coverage of key child health preventive practices in underserved populations through intensive community mobilization and routine, systematic household visitation [4,5]. Community-based strategies that foster frequent contact between caregivers of children under five years of age and credible sources of health information, including CHWs, are essential to achieve behavior change outcomes and sustained improvements in household practices [6,7]. However despite their potential, CHWs may face unmanageable workloads and large catchment areas, which limit their effectiveness for optimal behavior change [8]. In addition, while normative guidance exists, there is a lack of documented evidence on appropriate strategies to sustainably integrate various CHW cadres into the formal health system [9,10].

The Care Group Community Volunteer System is a community-based implementation strategy for the delivery of social and behavior change interventions [11]. The linchpin of this approach is the Care Group Volunteer (CGV); a woman living in the community who is elected by her peers and who herself has a child under the age of five years. The CGV participates in monthly meetings of her Care Group to receive lessons in a specific child health topic, and is then responsible for visiting her 10-12 neighbour households with interpersonal behavior change communication on the child health topic on at least a monthly basis. By collectively ‘saturating’ the target area, Care Groups have contributed to improved child health outcomes in a number of contexts [12,13].

Care Groups are facilitated by and through non-governmental organization (NGO) projects and project staff, and CGVs are trained and supervised by full-time project staff members called ‘Promoters’. Ministry of Health (MOH) leadership is necessary for scaling-up and sustaining community health initiatives [14]. Until this time, NGOs have not attempted to meaningfully collaborate with MOU counterparts to integrate the Care Group model into existing government delivery systems, with supervision of all Care Group activities remaining under the direction of full-time NGO staff [15].

We therefore tested the effect of replacing paid NGO supervisors with MOH supervisors who added Care Group supervision to their regular activities. Under this MOH-led Care Group model, CGVs are trained and supervised by a cadre of MOH-recognized CHWs, who are unpaid. The CHWs are in turn supervised by the head nurse at the health facility, who is in turn supervised by the District Health Team. The objective of the study was to determine if supervision of Care Group activities by MOH personnel could achieve the same results as supervision provided by specialized NGO staff.

## Methods

### Study design

The study was a pretest-posttest quasi-experimental design implemented in Burundi. The study was implemented in Bukinanyana Commune, which has a total population of approximately 110,000 and is one of three mountainous and rural communes in Mabayi Health District. Bukinanyana was selected in collaboration with district officials as the study location as it is the most populous and underserved of the three communes in the district, has no other NGOs active in the commune, and has the most limited availability of health services. The five zones within Bukinanyana Commune were grouped into two clusters based on population size (cluster one consisted of three zones and cluster two consisted of two zones). Each cluster was then randomly assigned by coin toss to either the intervention area (MOH-led Care Groups) or comparison area (NGO-led Care Groups). The intervention and comparison areas were contiguous. In the intervention area, 45 Care Groups with 478 CGVs were established. These MOH-led Care Groups were supervised by 26 CHWs, who were in turn supervised by the head nurse at each of the three health facilities within the intervention area. In the comparison area, 51 Care Groups with 503 CGVs were formed. These NGO-led Care Groups were supervised by six NGO supervisors. The head nurse at each of the four health facilities and the 34 CHWs within the comparison area were also oriented on the NGO-led Care Group model and encouraged to participate in Care Group activities.

### Implementation of the intervention

Care Group activities commenced in June 2011 in both study areas and were implemented according to Care Group standards [16] over a period of two years. Care Group meetings were held twice per month. In addition, CGVs were asked to conduct home visits to each of her 10-12 neighbouring households at least once per month, during which they provided health promotion messages, screened for acute malnutrition, and collected vital events data. In the MOH-led model, NGO staff first trained the District Health Team and health facility staff on the overall Care Group model and in the use of training materials with CHWs and CGVs. NGO staff also provided technical support as needed over the course of the study to the District Health Team and health facility staff to facilitate their leadership of Care Group activities.

Table 1 illustrates the key differences in MOH-led and NGO-led Care Group implementation. In the NGO-led model, the Promoter supervised between 5-9 Care Groups, which consisted of approximately 50-90 CGVs in total. The Promoter facilitated all Care Group meetings and was expected to supervise at least one CGV from each Care Group every month. Supervision consisted of accompanying the CGV on a household visit, reviewing her reporting forms, and providing feedback. In the MOH-led model, each CHW supervised between 1-3 Care Groups, which consisted of approximately 10-30 CGVs in total. The CHW facilitated all Care Group meetings and was also expected to supervise at least one CGV from each Care Group every month. The head nurse in turn supervised the CHW by attending at least one Care Group meeting per month, reviewing the CHW Care Group reporting forms and providing feedback. In addition, the head nurse facilitated a monthly meeting with all CHWs at the health facility, during which the CHWs submit their monthly Care Group reports and the nurses reviewed and provided support in resolving any problems related to Care Group implementation.

**Table 1:** Summary of Key Differences in NGO-led and MOH-led Care Group Models

	Comparison Area (NGO-led Care Groups)	Intervention Area (MOH-led Care Groups)
Ratio of Supervisors to Care Groups	1 Promoter: 5-9 Care Groups	1 CHW: 1-3 Care Groups

Training Methodologies	Promoters train Care Group Volunteers during the twice-monthly Care Group meetings	NGO staff provide quarterly one-day Training of Trainers to the District Health Team on three months’ worth of BCC modules. The District Health Team then cascades these trainings down through the MOH system as follows: • The District Health Team provides quarterly one-day trainings to the health facility nurses on the same modules • MOH nurses train the CHWs within their health facility catchment areas on those same topics on a monthly basis • CHWs provide that same training to their Care Groups during their twice-monthly Care Group meetings

Supervision	Care Group Volunteers are supervised by Promoters, who are in turn supervised by senior project staff	Supervision structure is through MOH system: • CHWs supervise Care Group Volunteers • CHWs are supervised by the nurse at each health facility

### Data collection methods

Quantitative data were collected before the intervention (September-November 2010) and after the intervention (June 2013) from among 593 and 700 mothers of children age 0-23 months, respectively. A census of all households with children 0-23 months in Bukinanyana Commune was conducted in July 2010 (baseline) and April 2013 (endline). The census identified 14,433 households with children 0-23 months, which constituted the sampling frame for the study and allowed us to take a simple random sample, with the household as the sampling unit. The baseline and endline sample size were calculated to detect a difference of 10% between the two intervention groups for each indicator being considered (one-sided alpha 0.05, beta 80%). Each randomly selected household was visited up to three times. The 16 enumerators at baseline consisted of project and MOH staff. Of the 21 enumerators at endline, 11 were project staff and 10 were external enumerators selected from the local community. Care was taken to ensure project staff were not serving as enumerators within their assigned project area.

### Intervention monitoring

Four process indicators were collected on a monthly basis to monitor Care Group activities in both study areas: the average number of Care Group meetings per month, CGV attendance at Care Group meetings, household visitation rates, and CGV reporting rates. In addition, a qualitative mid-term process evaluation was conducted to prospectively document the implementation and the functionality of the two Care Group models, as well as perceptions of the MOH-led and NGO-led models from MOH personnel and community members. A total of 15 focus group discussions (FGDs) were completed with mothers of children 0-23 months, Care Group Volunteers, CHWs, health facility nurses, Health Management Committee members, local leaders, and NGO Care Group supervisors (Promoters). The FGDs were grouped by participant category and by study area. In-depth interviews were also conducted with two NGO staff (Promoters) and one member of the District Health Team.

### Definition of variables

The study assessed changes in 40 child health knowledge and practice indicators, which are grouped into four main categories:

• **Preventive practices:** exclusive breastfeeding until six months, hand-washing at critical times, use of insecticide-treated bed net

• **Knowledge:** knowledge of childhood illness danger signs, complementary feeding, and proper care of a sick child

• **Care-seeking practices for child illness:** care-seeking and treatment for children sick with diarrhoea, fever, and acute respiratory infections

• **Contact intensity:** contact with trained provider of health information and participation in meetings during which child health is discussed

We sought to test whether the MOH-led Care Group model would achieve *at least* the same coverage of the knowledge and practice of key health and nutrition behaviors as the NGO-led model after two years of implementation (June 2011-May 2013).

### Analysis techniques

We used Pearson’s chi-squared test to evaluate whether there was a statistically significant difference in endline indicators in the intervention and comparison areas. Since this study was quasi-experimental, we also conducted a difference-in-differences (DiD) analysis [17,18]. To do so, we calculated the coverage rates for each of the indicators in 2010 and 2013 in both the intervention and comparison areas. The percentage increase in coverage in comparison areas was then subtracted from the percentage increase in intervention areas to obtain the difference in the differences; as summarized by the formula below:

where  and  represent the average outcome at endline in the intervention site and comparison area, respectively, and  and  the average outcome at baseline in the intervention site and comparison area, respectively. Given our hypothesis that the MOH-led model performs at least as well as the NGO-led model in the achievement of key child health indicators, both the chi-squared and DiD analysis specifically sought to assess if indicators in the intervention area perform statistically significantly *worse* than those in the comparison area.

### Ethical considerations

Ethical approval for this study was obtained from the Trinity College of Dublin Health Policy and Management’s Centre for Global Health Research Ethics Committee. Informed consent was verbally obtained from each respondent prior to participating in the baseline and endline surveys as well as the FGDs and interviews during the midterm process evaluation, as the majority of respondents did not have sufficient literacy skills to provide written consent.

## Results

### Respondent characteristics

Table 2 presents the demographic and socio-economic characteristics of households with children aged 0-23 months in the intervention and comparison areas at baseline. Respondents in both areas were equivalent in levels of education and household composition, but there were statistically significant differences in three household economy measures: household dietary diversity, the per cent of households that work outside the home to earn money, and the per cent of households that sell their agricultural goods at the market. These indicators paint a mixed picture of economic status in the intervention and comparison groups: a more diversified diet is correlated with higher household income [19], suggesting that the comparison area might have been wealthier. This is reinforced by the fact that providing casual labor on other people’s farms, which was more prevalent in the intervention area, is usually an activity in which poorer households are engaged and is sometimes considered a coping strategy [20]. Significantly more households in the comparison area sold their crops at local markets, and while households who sell cash crops are generally better off than those who rely on subsistence farming, it may also be that households sold their crops to earn cash for other non-food items; which would also be considered a coping strategy [21]. Differences in these indicators between the two study areas did not persist at endline.

**Table 2:** Demographic and Socio-economic Characteristics of Households with Children Age 0-23 Months at Baseline

Characteristic	Comparison Area (NGO-led Model) N = 296	Intervention Area (MOH-led Model) N = 297	p-value
		
	%	%	
**Child’s Sex**			

Male	52.0	54.5	.539

Female	48.0	45.5	.595

**Child’s Age**			

0-5 months	14.9	9.8	.075

6-11 months	25.1	29.0	.333

12-23 months	60.0	61.2	.815

**Household Composition**			

Mean # of children 0-23 months	1.9	1.8	.297

**Mother’s Level of Education**			

Illiterate	66.2	67.8	.862

Basic literacy	27.0	26.9	.926

Completed primary school	6.8	5.3	.486

**Household Economy**			

Mean household dietary diversity score (total of 11 food groups)	4.65	4.2	.004**

Woman does agricultural work on other's land to earn money	17.9	29.3	.002**

Woman sells crops at market to earn money	28.7	16.8	.001***

**Abbreviations:** MOH, Ministry of Health; NGO, non-governmental organization

**Notes:** *p<.05 ** p<.01 ***p<.001

### Intervention monitoring

Care Groups in both the intervention and comparison areas functioned similarly throughout the study period. An average of 1.9 Care Group meetings was held per month in both study areas, largely meeting the standard of two meetings per month. Care Group Volunteer attendance was consistently high: 87% of NGO-led Care Groups and 91% of MOH-led Care Groups met the target of at least 80% CGV attendance in at least one meeting each month. NGO-led Care Groups achieved slightly higher home visitation rates, with an average of 94% of households receiving at least one visit from a CGV per month, compared with 87% in the MOH-led Care Groups. Care Group Volunteer reporting rates were the same in both study areas, with an average of 96% of CGVs submitting reports to their respective supervisor each month.

The mid-term process evaluation revealed two important findings related to Care Group implementation in both study areas. The first finding is related to the role of CHWs under the NGO-led Care Group model: at the beginning of the intervention, project staff oriented both health facility personnel and CHWs to the Care Group concept and encouraged their participation in Care Group activities. The process evaluation found that instead of merely participating in Care Group activities in the comparison area, CHWs were taking a more active role in Care Group meeting facilitation and reporting, effectively serving as assistants to the NGO supervisor. The process evaluation also found that, in the intervention area, supervision from the health facility level was not working as planned. In the original intervention design, the head nurse at each health facility was tasked with overseeing all Care Group activities within the health facility catchment area, through the direct supervision of the CHWs. However, the head nurse had many other responsibilities at the health facility and with the District Health Team, which left them little time to oversee community level activities. As stated by one head nurse in the intervention area, “The workload has increased a lot for the *Titulaires* [head nurses] because we have lots to do. If we add this [Care Group supervision] to our other responsibilities we will not be effective. Really, we don’t have the time to do supervision of these activities.” As such, a junior nurse at each health facility, who had fewer responsibilities and therefore had more time to supervise CHWs and their respective Care Group activities, was identified.

### Child health indicators

Chi-squared testing found that 38 of the 40 indicators performed at least as well in the intervention area as in the comparison area. The DiD analysis, which accounts for difference s in baseline values, found that the MOH-led model performed at least as well as the NGO-led model in 39 of 40 outcome indicators. Supplementary Tables 1 and 2 (additional files 1 and 2) provide results for all 40 indicators. In the interest of space, we report on a sub-set of 10 key dependent variables, which have a robust causal association with child mortality [22,23].

Table 3 presents the results of the chi-squared testing for the 10 key child health practices in the intervention and comparison areas at endline. Endline values for all 10 indicators were at least as high for the intervention area as the comparison area; and were statistically significantly higher for two indicators (care-seeking for all illnesses and hand-washing at critical times).

**Table 3:** Key Child Health Indicators across Intervention and Comparison Areas

Indicator		Comparison Area (NGO-led Model)		Intervention Area (MOH-led Model)		p-value
		
		N	%	N	%	
Exclusive breastfeeding	Percent of children age 0-5 months who consumed only breast milk within the 24 hours preceding the survey	51	92.2	59	91.5	.904

Dietary diversity	Percent of breastfed children age 6-23 months who consumed foods from four or more food categories (from a total of eight) within the 24 hours preceding the survey	267	52.1	263	55.9	.279

Knowledge of complementary feeding	Percent of mothers of children age 0-23 months who correctly identify six months as the appropriate age at which to introduce any other foods or liquids other than breast milk	347	86.5	353	90.1	.135

ORT use	Percent of children age 0-23 months with diarrhea in the two weeks preceding the survey who received ORS and/or recommended home fluids	66	89.4	53	92.5	.567

Prompt anti-malarial treatment	Percent of children age 0-23 months with fever in the two weeks preceding the survey who were treated with an effective anti-malarial drug within 24 hours after the fever began	159	17	166	20.5	.419

ARI care-seeking and treatment	Percent of children age 0-23 months with cough and fast/difficult breathing in the two weeks preceding the survey who were taken to a health facility or received antibiotics from an alternative source	154	88.3	143	92.3	.246

Care-seeking all illnesses	Percent of children age 0-23 months with diarrhea, fever, or ARI symptoms in the two weeks preceding the survey whose caregivers sought care or advice outside the home	234	86.3	229	93.4	.011*

Hand washing at critical times	Percent of caregivers of children age 0-23 month who reported washing their hands with soap during at least three of four critical times (before preparing food, before feeding a child, after defecation, after attending to a child who has defecated) in the previous 24 hours	347	22.8	352	34.7	.001***

ITN use	Percent of children age 0-23 months who slept under an ITN the previous night	344	32	352	34.9	.407

Contact intensity	Percent of mothers of children age 0-23 months with at least two personal contacts with a trained provider or health information in the previous month	347	77.5	353	79.3	.563

**Abbreviations:** ARI, acute respiratory infection; ITN, insecticide-treated bed net; MOH, Ministry of Health; NGO, non-governmental organization; ORT, oral rehydration therapy.

**Notes:** *p<.05 ** p<.01 ***p<.001

Table 4 presents the DiD analysis, which takes into account differences in all 10 indicators at baseline. There was a strong positive effect of the intervention on one indicator (hand washing at three of four critical times). Although the DiD results suggest variable positive and negative effects of the intervention in the other nine indicators, none of the other results was statistically significant. The DiD analysis therefore demonstrates that the MOH-led model performed at least as well as the NGO-led model in all 10 outcome indicators.

**Table 4:** Difference-in-Differences Analysis for Key Child Health Indicators

Indicator	Comparison Area (NGO-led Model)					Intervention Area (MOH-led Model)					Difference in the Differences
		
	Baseline (2010)		End line (2013)		Change	Baseline (2010)		End line (2013)		Change	
	
	N	%	N	%	%	N	%	N	%	%	%
Exclusive breastfeeding	44	36.4	51	92.2	**+55.8**	29	51.7	59	91.5	**+39.8**	**-16**

Dietary diversity	246	52.4	267	52.1	**-0.3**	266	45.1	263	55.9	**+10.8**	**+11.1**

Knowledge of complementary feeding	296	69.9	347	75.0	**+5.1**	296	86.5	353	90.1	**+3.6**	**-1.5**

ORT use	88	85.2	66	89.4	**+4.2**	93	78.5	53	92.5	**+14**	**+9.8**

Prompt anti-malarial treatment	90	12.2	159	17.0	**+4.8**	96	11.5	166	20.5	**+9**	**+4.2**

ARI care-seeking and treatment	80	75.0	154	88.3	**+13.3**	65	80.0	143	92.3	**+12.3**	**-1**

Care-seeking all illnesses	149	77.2	234	86.3	**+9.1**	162	78.4	229	93.4	**+15**	**+5.9**

Hand washing at critical times	296	7.8	347	22.8	**+15**	296	7.4	352	34.7	**+27.3**	+12.3**

ITN use	262	80.9	344	32	**-48.9**	263	78.7	352	34.9	**-43.8**	**+5.1**

Contact intensity	296	5.4	347	77.5	**+72.1**	296	8.8	353	79.3	**+70.5**	**-1.6**

**Abbreviations:** ARI, acute respiratory infection; ITN, insecticide-treated bed net; MOH, Ministry of Health; NGO, non-governmental organization; ORT, oral rehydration therapy.

**Notes:** ** p<.01

## Discussion

### Summary of findings

We found that the MOH-led Care Group model performed at least as well as the NGO-led model in achieving specific child health and nutrition outcomes. Mothers of children 0-23 months in the intervention and comparison sites reported similar levels of knowledge and practices for 38 of 40 dependent variables measured in the study, and these results remained unchanged after accounting for differences in the indicator values at baseline. Process monitoring data confirmed that the MOH-led Care Group model and the NGO-led Care Group model were implemented with similar intervention strength.

### Implications for the scale-up of community-based Iiterventions through MOH systems

Ministries of Health in several countries have adopted large-scale community health initiatives that include a role for one or two CHWs in each village to generate demand for health services and expand coverage of key child health preventive practices. [24-26]. However, even relatively small villages have target populations that make it difficult for one volunteer CHW to realistically conduct monthly household visitation, especially in settings like Burundi, where nearly 20% of the total population is under five years of age [27]. The MOH-led Care Group model demonstrates how CGVs are able to extend the reach of CHWs to achieve high coverage of interpersonal communication required for behavior change through routine, systematic household visitation through existing MOH systems. With each CGV responsible for up to 12 households, and approximately 10 CGVs in one Care Group, the power of one CHW is multiplied to reach up to 120 households through the Care Group.

In addition to achieving higher coverage levels, the MOH-led Care Group model builds the capacity of existing CHWs, thereby strengthening human resources within the overall health system. As CHWs train and supervise CGVs, they are simultaneously strengthening their own leadership and technical skills. Moreover, CHWs gain a higher status in the community as they engage in supervisory activities comparable to NGO staff. The fact that CHWs organically took on a more active role in Care Group meeting facilitation and reporting in the NGO-led model also speaks to CHWs’ skills and leadership potential.

In the MOH-led Care Group model, the primary actors responsible for facilitating Care Group activities are existing health workers within the MOH system. However, NGO staff still have a critical role to play in building the capacity of the MOH to implement the model. In the intervention area, NGO staff provided initial training to the District Health Team and health facility staff on the overall concept of Care Groups, facilitated the population census and election of CGVs, conducted formative research to inform the design of the behavior change modules used by CGVs, developed the CGV reporting tools, and provided quarterly training-of-trainers to the District Health Team on the content of those modules. As the NGO continues to support MOH counterparts to implement the Care Group model in additional districts and provinces, many of these responsibilities could be taken on by MOH actors over time. For the MOH-led model to be truly integrated into national community health systems, additional advocacy and capacity building on the model will be required at the national level.

### Implications for program supervision

The MOH-led Care Group model includes a two-tier supervision structure. The CGVs are supervised by CHWs, who are in turn supervised by the assistant nurse at the health facility. CHWs reported that the additional Care Group supervision tasks *lightened* their work load, as the multiplying power of Care Groups enabled the CHW to effectively delegate the household visitation tasks to the CGVs. This is particularly important in countries where CHWs are increasingly asked to take on additional responsibilities beyond household visitation, such as community case management.

Another key finding of the study was the fact that the junior as well as senior nurses were able to supervise Care Groups. While it may be difficult for the MOH to assume Care Group supervision responsibilities if scarce mid-level human resources are required, prospects may be better if these responsibilities are tasked to under-utilized lower level human resources, such as the assistant nurses. It is possible that we overestimated the importance of mid-level human resources to supervise community-level activities; studies of task shifting in low-income countries’ programs have found that lower level cadres of health workers are able to deliver and supervise quality and comparable health education, promotion, and care to communities when compared with clinical providers [28,29].

For an effective implementation strategy to be scaled up, it must be cost effective and feasible for the health system to maintain. A previous evaluation of an NGO-led Care Group project had an average cost of $2.78 per beneficiary per year [30]. While an assessment of cost effectiveness is beyond the scope of this paper, the MOH-led Care Group model does provide some cost savings to the NGO model in terms of supervisor staff salary, recruitment and transportation expenses. Our study demonstrates that the MOH-led Care Group model may be implemented using existing and under-utilized human resources within the MOH system. Therefore, Care Group supervision costs borne by the MOH would therefore most likely be the opportunity costs of asking existing health workers to assume additional responsibilities and/or shift some responsibilities to lower level cadres. Future studies are required to systematically document all inputs and monetary costs borne by the MOH to implement the model and compare the cost effectiveness of the NGO and MOH-led models.

### Limitations

Statistically significant differences in socio-economic status between the study areas at baseline may have confounded the results, although the differences did not persist at endline. These differences may be partially explained by the fact that the study design was quasi-experimental rather than experimental, or that the baseline and endline surveys were conducted at different times of the year, October 2010 and May 2013, respectively. The rainy season lasts through October, which extends the hunger period, as food stocks are depleted and crops are not yet ready for harvest. There is usually an increase in malnutrition and malaria cases in October. Therefore, it is likely that the endline survey occurred when households were relatively better off. This does not affect the chi-square findings since this analysis was only done at endline, however it may have affected the difference-in-differences results. Using project staff and MOH staff as enumerators may also have biased respondent results, however care was taken to mitigate this bias by assigning enumerators to areas outside of their assigned project area.

## Conclusion

The results presented in this paper point to the potential of an MOH-led Care Group model to overcome human resource constraints to achieve high coverage of community-based health interventions through existing government health systems. To fully assess the potential for sustainability of the MOH-led model, an additional study is required to document the extent to which the MOH continues to support Care Group implementation as well as whether results are sustained in both the NGO-led and MOH-led Care Group areas. In addition, future research assessing what other typical NGO inputs could be shifted to the MOH and the implications of those shifts for the scale-up and sustainability of community health interventions is needed. The MOH in Burundi, specifically the District Health Team, was actively involved in designing the implementation of the MOH-led Care Group model, and future implementers should collaborate with MOH counterparts to assess the most optimal way that Care Groups can be integrated into MOH systems.

## List of abbreviations

CGV: Care Group Volunteer; CHW: Community Health Worker; DiD: Difference in Differences; FGD: Focus Group Discussion; MOH: Ministry of Health; NGO: Non-Governmental Organization

## Competing interests

The authors declare no competing interests.

## Authors’ contributions

JW participated in the study’s design and coordination and drafted the manuscript. RM conducted all statistical analysis and helped to draft the manuscript. DS conceived of the study and participated in its design and coordination. All authors read and approved the final manuscript.

## Supplementary Material

Additional file 1 Click here for file

Additional file 2 Click here for file

## Declarations

The publication costs for this article were funded by a grant from by the Office of Health, Infectious Disease and Nutrition; Bureau of Global Health, United States Agency for International Development (USAID). The contents of this article are the responsibility of the authors and do not reflect the views of USAID or the United States Government.

This article has been published as part of *BMC Health Services Research* Volume 15 Supplement 1, 2015: Improved access to maternal, newborn and child health services: strengthening human resources for health. The full contents of the supplement are available online at http://www.biomedcentral.com/bmchealthservres/supplements/15/S1.
