# Supplementary material for: Shifting management of a community volunteer system for improved child health outcomes: results from an operations research study in Burundi
Source: BMC Health Serv Res. 2015 Jun 8;15(Suppl 1):S2. doi: 10.1186/1472-6963-15-S1-S2 (PMC4464208; doi:10.1186/1472-6963-15-S1-S2)
Supplement: Additional file 1 [file 1472-6963-15-S1-S2-S1.docx]

| **Supplementary Table 1: Endline Chi-Squared Analysis for all Variables** | | | | | | |
| --- | --- | --- | --- | --- | --- | --- |
| **No.** | **Indicator** | **Comparison Area (NGO-led Model)** | | **Intervention Area (MOH-led Model)** | | **p-value** |
|  |  | **N** | **%** | **N** | **%** |  |
| *Preventive Practices* | | | | | | |
| 1 | Percent mothers of children age 0-23 months who took iron tablets before the birth of their youngest child | 347 | 95 | 353 | 92.3 | .228 |
| 2 | Percent mothers of children age 0-23 months who received at least 90 days of iron-folate supplementation during their most recent pregnancy | 347 | 19.7 | 353 | 16.9 | .415 |
| 3 | Percent children age 0-23 months who were put to the breast within one hour of delivery | 347 | 87.2 | 353 | 91.6 | .064 |
| 4 | Percent children age 0-23 months who were fed colostrum after birth | 347 | 100 | 353 | 100 | 0 |
| 5 | Percent children age 0-5 months who were exclusively given breast milk the day prior to the interview | 51 | 92.2 | 59 | 91.5 | .904 |
| 6 | Percent breastfed children age 6-23 months who ate from three or more food categories (from total of eight) during previous 24 hours | 267 | 67.4 | 263 | 77.9 | .005** |
| 7 | Percent breastfed children age 6-23 months who ate from four or more food categories (from total of eight) during previous 24 hours | 267 | 52.1 | 263 | 55.9 | .279 |
| 8 | Percent children age 6-23 months fed a solid or semi-solid food with the recommended frequency for their age group/ breastfeeding status during previous 24 hours | 296 | 37.5 | 294 | 44.2 | .097 |
| 9 | Percent children age 6-23 months who ate an animal source food during previous 24 hours | 296 | 38.2 | 294 | 44.2 | .136 |
| 10 | Percent mothers of children age 0-23 months who live in households with soap at the place for hand washing | 347 | 91.6 | 353 | 94.9 | .087 |
| 11 | Percent mothers of children age 0-23 who reported washing their hands with soap during at least three of four critical times the previous 24 hours | 347 | 22.8 | 353 | 34.7 | .001*** |
| 12 | Percent children age 0-23 months whose household owns an insecticide-treated bed net | 347 | 51.9 | 353 | 54.4 | .505 |
| 13 | Percent children age 0-23 months who slept under an insecticide-treated bed net the previous night | 347 | 32 | 353 | 34.9 | .407 |
| *Knowledge of Healthy Behaviors* | | | | | | |
| 14 | Percent mothers of children age 0-23 months who knew at least two signs of childhood illness that indicate the need for treatment (looks unwell or not playing normally, not eating or drinking, lethargic or difficult to wake, high fever, fast or difficult breathing, vomits everything, convulsions). | 347 | 91.1 | 353 | 89.0 | .351 |
| 15 | Percent mothers of children age 0-23 months who know the importance of increasing food intake during episodes of diarrhea | 347 | 58.5 | 353 | 52.1 | .090 |
| 16 | Percent mothers of children age 0-23 months who can name at least three of the four most critical times to wash hands with soap (before food prep, before feeding children, after defecation, after attending to a child who has defecated) | 347 | 53.9 | 353 | 69.1 | .000*** |
| 17 | Percent mothers of children age 0-23 months who know that additional food intake is required during pregnancy | 347 | 54.2 | 353 | 56.1 | .611 |
| 18 | Percent mothers of children age 0-23 months who know iron-foliate tablets are essential during pregnancy | 347 | 91.2 | 353 | 98.3 | .000*** |
| 19 | Percent mothers of children age 0-23 months who know a child should be put to the breast within the first 3 hours of birth | 347 | 99.4 | 353 | 99.7 | .553 |
| 20 | Percent mothers of children age 0-23 months who can identify six months as the appropriate age at which to introduce any food or liquid other than breast milk | 347 | 86.5 | 353 | 90.1 | .135 |
| 21 | Percent mothers of children age 0-23 months who can name a list of foods that make up a well- balanced diet (energy dense foods, micronutrient dense foods and strength giving foods) for a child older than 6 months of age | 347 | 90.8 | 353 | 96.9 | .001** |
| 22 | Percent mothers of children age 0-23 months who can name at least three energy-dense locally available foods to feed their child each day | 347 | 39.2 | 353 | 43.8 | .239 |
| 23 | Percent mothers of children age 0-23 months who can name at least three micronutrient-rich foods to feed their child each day | 347 | 20.9 | 353 | 17.4 | .284 |
| 24 | Percent mothers of children age 0-23 months who can name at least three strength building foods to feed their child each day | 347 | 30.4 | 353 | 34.0 | .357 |
| 25 | Percent mothers of children age 0-23 months who know the appropriate frequency of meals/snacks for a child 6-8 months of age | 347 | 29.1 | 353 | 22.4 | .042* |
| 26 | Percent mothers of children age 0-23 months who know the appropriate frequency of meals/snacks for a child 9-23 months of age | 347 | 61.7 | 353 | 52.7 | .016* |
| *Sick Child Care-Seeking Practices* | | | | | | |
| 27 | Percent children age 0-23 months with diarrhea in the last two weeks who received oral rehydration solution (ORS) and/or recommended home fluids (RHF) | 109 | 89.4 | 89 | 92.5 | .567 |
| 28 | Percent children age 0-23 months with diarrhea in the last two weeks who were offered more fluids during the illness | 109 | 60.6 | 89 | 75.3 | .028* |
| 29 | Percent children age 0-23 months with diarrhea in the last two weeks who were offered the same amount or more food during the illness | 109 | 44.9 | 89 | 44.3 | .940 |
| 30 | Percent children age 0-23 months with diarrhea in the last two weeks whose mothers sought outside advice or treatment for the illness | 109 | 81.7 | 89 | 86.5 | .355 |
| 31 | Percent children age 0-23 months with a febrile episode during the last two weeks who were taken to an appropriate place for treatment | 159 | 99.3 | 166 | 98.7 | .611 |
| 32 | Percent children age 0-23 months with a febrile episode during the last two weeks who were treated with an effective anti-malarial drug within 24 hours after the fever began | 159 | 17.0 | 166 | 20.5 | .419 |
| 33 | Percent children age 0-23 months with a febrile episode during the last two weeks who were treated with ACT within 24 hours after the fever began | 159 | 10.7 | 166 | 13.9 | .386 |
| 34 | Percent children age 0-23 months with a febrile episode during the last two weeks who were treated with an effective anti-malarial drug | 159 | 18.9 | 166 | 22.3 | .446 |
| 35 | Percent children aged 0-23 months with cough and fast/difficult breathing in the last two weeks who were taken to a health facility or received antibiotics from an alternative source | 154 | 88.3 | 143 | 92.3 | .246 |
| 36 | Percent all sick children age 0-23 months who sought care or advice outside home | 234 | 86.3 | 229 | 93.4 | .011* |
| *Contact Intensity* | | | | | | |
| 37 | Percent mothers of children age 0-23 months with at least one personal contact with a trained provider of health information within the last month | 347 | 88.2 | 353 | 93.5 | .228 |
| 38 | Percent mothers of children age 0-23 months with at least two personal contacts with a trained provider of health information within the last month | 347 | 77.5 | 353 | 79.3 | .563 |
| 39 | Percent mothers of children age 0-23 months who attended any type of meeting in the community at least once in which her health or the health of her child was discussed. | 347 | 34.0 | 353 | 37.7 | .344 |
| 40 | Percent mothers of children age 0-23 months who attended any type of meeting in the community at least twice in which her health or the health of her child was discussed. | 347 | 19.6 | 353 | 18.4 | .690 |
|  | **Abbreviations:** ARI, acute respiratory infection; ITN, insecticide treated bed net; MOH, Ministry of Health; NGO, non-governmental organization; ORT, oral rehydration therapy.  **Notes:** *p<.05 ** p<.01 ***p<.001 | | | | | |
